# A Clinical Case with Appendices

**Published:** 1888-03

**Authors:** J. N. Lowe

**Affiliations:** Milford, N. J.


### A CLINICAL CASE WITH APPENDICES.

J. N. LOWE, M. D., MILFORD, N. J.

E. F., a boy, æt. twelve years—residing seven miles distant—was brought to my office by his father, desiring to know the nature of his indisposition. The day was cold, and an icy, damp, chilling atmosphere prevailed.

It required but casual observation to decide the cause of his illness. He had scarlet fever, anginosa, and his symptoms presented a very considerable degree of severity. The characteristic eruption was well expressed; pulse 140; respiration greatly accelerated; throat highly inflamed; deglutition very painful and difficult; breath very offensive; submaxillary glands enlarged; larynx painful; and a bad discharge issued from his nose.

I scarcely took time to consider the ensemble of his symptoms, but hurried his father away with him, and giving him a few powders of Belladonna, I promised to see him at his home the following morning.

When I visited him I found an aggravation of all the most unfavorable symptoms and an amelioration of none in particular, and at once came to the conclusion that the septic influence was becoming rapidly dominant, and that something must be done to change the current of morbid affairs.

I thought of Dr. Lippe's portrayal of physiological and pathological picture books. Having always believed that the excellent doctor's criticisms of these phantom books were at the least practically correct, at the conclusion of this case I had no difficulty in deciding that he is entirely true in his denunciatory dicti of these fanciful volumes.

"The Law" said, Give your patient Lycopodium. Scientific physician (!) and pathologist said, *No!* do something material. Prescribe from a physiological and pathological standpoint. Beard the lion in his den. You have no time to lose.

Eclecticism said: Apply kerosene and camphor to the throat; cover it with bacon; use gargles and sprays; administer the most fashionable and efficient antiseptic medicines—in a word, do all that a Jupiter Maximus should do; thus you will destroy the pesky bacteria b., and all will result—well.

We refused to accede to the dictum of the latter Hercules, rather preferring to give the "very pest remety" in the materia medica, to wit:—Sac. lac—than to become a murderous slayer of indefensible bacteria; as they have never yet to our knowledge done our patients or ourselves any harm. We administered Ars. iodid.[6]; at three hour intervals, for twenty-four hours. This remedy seemed to cause a stir in the pathological quarters, and we inferred that it had been of some service, as the nasal discharge was somewhat improved in character and other putrescent manifestations were somewhat modified; but the force of the disease was more than ever concentrated in the larynx, which caused us to change to Bromium[3]. The throat was filled with putrid ulcers and foul secretions. Continuing Bromium for twenty-four hours, it seemed to produce some amelioration of the laryngeal trouble; however, the key to the arch of morbid symptoms remained intact, and had thus far defied the potency of our art to dislodge it. All that we could claim to have accomplished was that we had checked the enemy in his aggressive march, while he yet continued to flaunt the black flag in our face.

It is strange that it becomes so difficult to suppress the predilections and the promptings instituted by custom and education when they conflict with our better convictions of truth. For centuries it has been taught *ex cathedra*, viz.: that symptomatology should always be regarded as of minor importance, and that it should never be permitted to stand in the way of a venerated system of physiology and pathology, upon which the treatment of all diseases should be based.

The results of this clinical case, which is not in the least exceptional or remarkable, will add one more to innumerable other similar examples which have proved and must ever continue to prove in the same sort of mastery, viz.: that symptoms furnish the only rational reflection and language of disease, including in their ensemble all that is usually prefigured in its most accurate pathology. However, pathology essentially aids us in our diagnosis and nosological classification of diseases and in prognosis; as to the influence, manifested by symptoms, upon normal or abnormal tissue-changes, expectant or actually occurring, and, very decidedly, as to what will be the ultimate result of such changes.

We by no means claim that we can dispense with pathology in the construction of the edifice of truthful, philosophic medicine. We do not present a system of disjointed fractions, but the whole fitly framed and joined together.

Symptomatology, pathology, and therapy are joined hand in hand. Pathological requirements in a remedial sense are squarely met by an accurate interpretation of symptoms, which unfailingly indicate the true simillimum, and as we progress in our accurate measurements our pleasure will increase as we witness the fading out of pathological terrors when confronted by nature's law.

Again, if we desire to engage in orthodox sentimentalism, and in theoretical and empirical romance, we should begin at the other end and fiddle, even if Rome does burn; for by the persistent drawing of the bow (at a venture) we may by and by strike the keynote of the pathological arch, when something similar will happen, and the old axiomatic saying, viz.: that "guesswork is as good as any when it hits," will again and again be revived.

We do not claim that mechanical and chemical morbid causes of diseased action can be removed by dynamized remedies. We trust that we have never yet fallen into such a realm of fatuity. But we do know, and we decidedly declare, that we have time and again routed and dispelled the dreaded diphtheritic germs in apparently malignant cases with the 200th and, still better, with the 1,000th centesimal characteristic similar. Furthermore, this plan has been so satisfactory (for which special thanks are due to Dr. Gregg) that I should never dare to use a low or medium potency, since the medium high has given such gracious results. I do not insert this paragraph as a fulsome or special plea for high potencies, nevertheless, these transcendental friends have kept me on my feet, time and again, in dire extremes, when the central organic nervous system has been deeply invaded by the emissaries of death, in the various examples of disease, after I have sustained defeat and been "knocked out" while wielding implements in the decimal and lower centesimal scales.

Can you blame me, gentlemen, for standing *hard by* my friends?

Truth will not conform to our prepossessed predilections and prejudices. We must accept it as we find it, or be shamed and crushed by it.

To resume our narration: Our patient's throat continued worse on the right side, and was so at the beginning. He objected to cold drinks; the motion of the alæ nasi was valvular; his mind and disposition were tinctured with ill-humor; he awoke from his delirious slumbers unpleasant and cross; his bowels were constipated. At this stage of his illness he received one powder Lycopodium$^{1m}$, dry, and was ordered three more powders of the same, to be taken in the ensuing twenty-four hours.

Tepid sponge-baths were ordered to be repeated from time to time, whenever a high cutaneous temperature, delirium, and restlessness should be dominant.

Lycopodium in the 1m quickly changed the complexion of this unpromising case, and a happy convalescence promptly ensued. Farther on a few doses of Lach.$^{1m}$ was given for cardiac disturbances and weakness. Lastly, a few doses of Sulphur$^{1m}$ in the process of desquamation.

Four other children of this family contracted this fever in course. One of these had it in the severest anginosa form, in which *Bryonia*$^{200}$ proved the true similar. All recovered. We rejoice that with the true, the pure homœopathic simillimum, given high (for we have very little faith in low potencies and in low dilutions in all dangerous cases of disease of dynamic origin, wherein the vegetative nervous system and its centres are profoundly affected and vitally depressed), we may penetrate the enemy's camp, even at the seeming last, rout the life-robbing Barabbas, draw down his ghastly ensign, change apparent defeat into victory, and, the best of all, cheer the hearts and dry the tears of a grieved and affectionate family.

